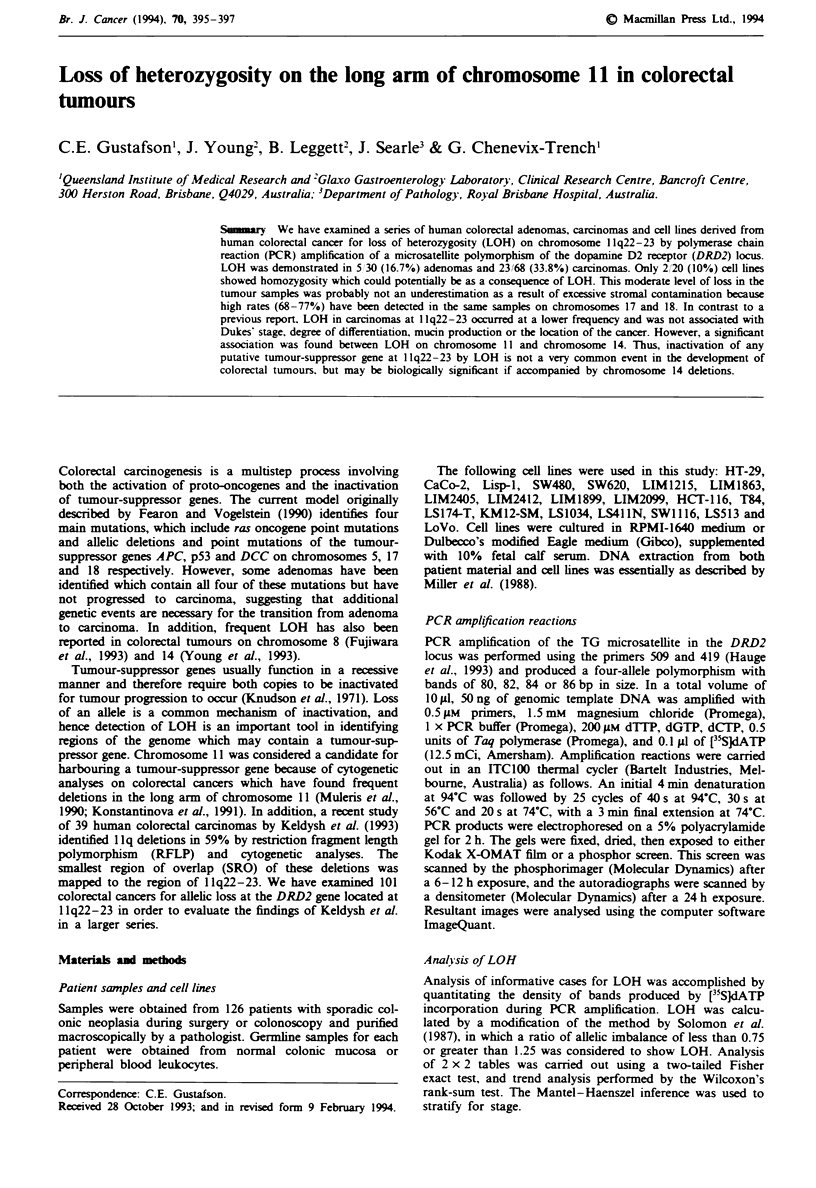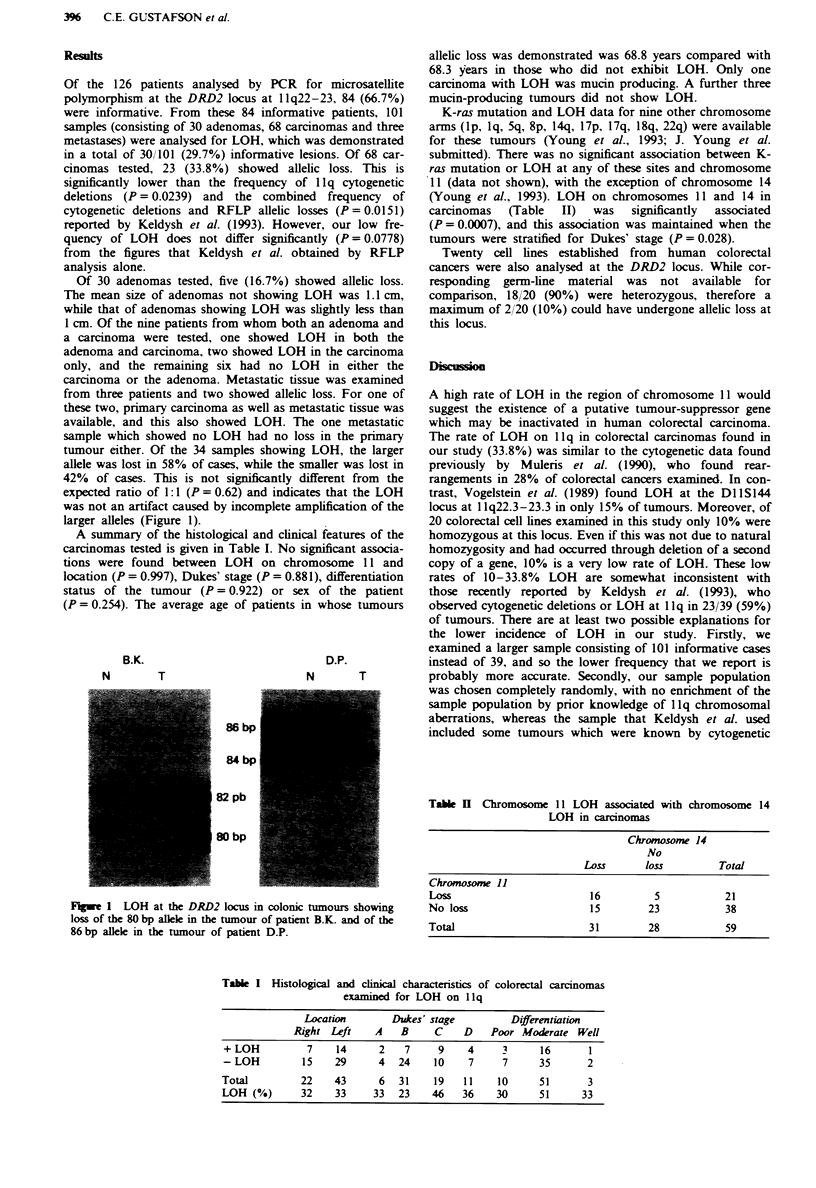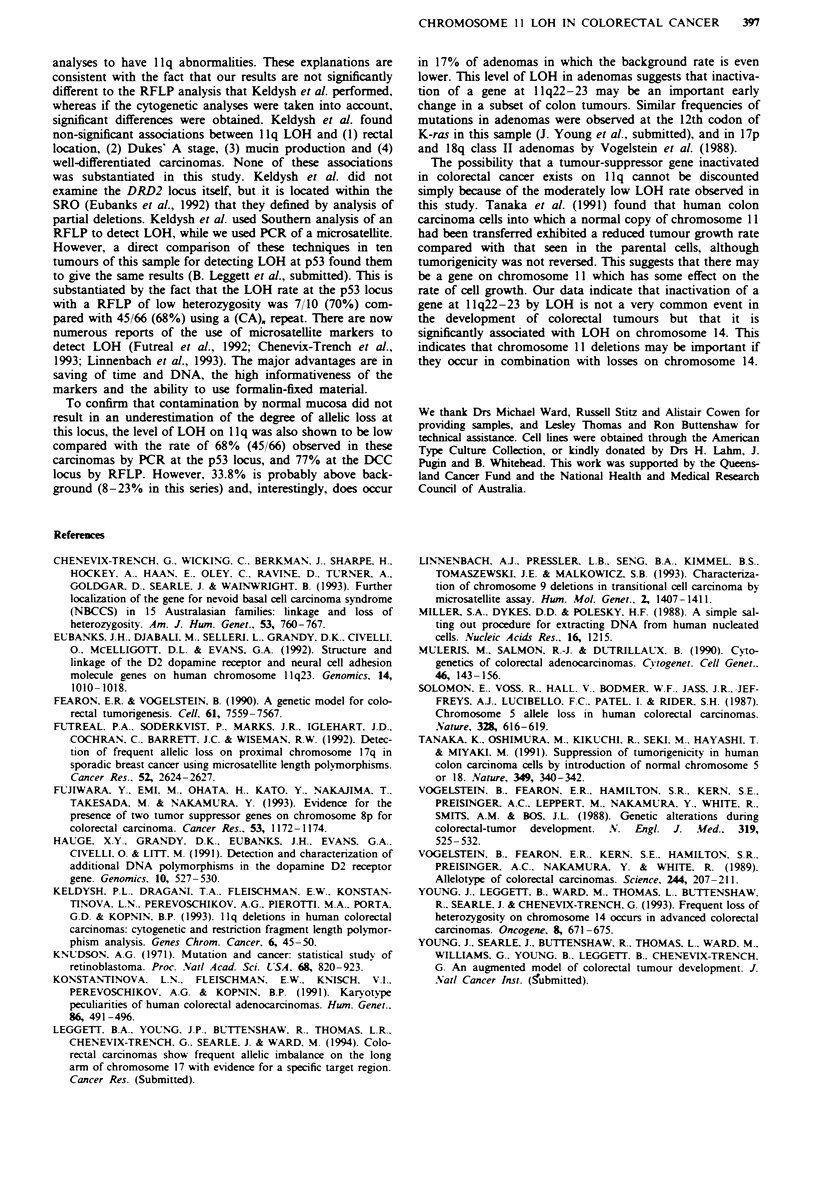# Loss of heterozygosity on the long arm of chromosome 11 in colorectal tumours.

**DOI:** 10.1038/bjc.1994.315

**Published:** 1994-09

**Authors:** C. E. Gustafson, J. Young, B. Leggett, J. Searle, G. Chenevix-Trench

**Affiliations:** Queensland Institute of Medical Research, Brisbane, Australia.

## Abstract

**Images:**


Br. J. Cancer (1994), 70, 395–397 © Macmillan Press Ltd., 1994

### Loss of heterozygosity on the long arm of chromosome 11 in colorectal tumours

C.E. Gustafson[1], J. Young[2], B. Leggett[2], J. Searle[3] & G. Chenevix-Trench[1]

[1]*Queensland Institute of Medical Research and* [2]*Glaxo Gastroenterology Laboratory, Clinical Research Centre, Bancroft Centre, 300 Herston Road, Brisbane, Q4029, Australia;* [3]*Department of Pathology, Royal Brisbane Hospital, Australia.*

**Summary** We have examined a series of human colorectal adenomas, carcinomas and cell lines derived from human colorectal cancer for loss of heterozygosity (LOH) on chromosome 11q22–23 by polymerase chain reaction (PCR) amplification of a microsatellite polymorphism of the dopamine D2 receptor (*DRD2*) locus. LOH was demonstrated in 5/30 (16.7%) adenomas and 23/68 (33.8%) carcinomas. Only 2/20 (10%) cell lines showed homozygosity which could potentially be as a consequence of LOH. This moderate level of loss in the tumour samples was probably not an underestimation as a result of excessive stromal contamination because high rates (68–77%) have been detected in the same samples on chromosomes 17 and 18. In contrast to a previous report, LOH in carcinomas at 11q22–23 occurred at a lower frequency and was not associated with Dukes' stage, degree of differentiation, mucin production or the location of the cancer. However, a significant association was found between LOH on chromosome 11 and chromosome 14. Thus, inactivation of any putative tumour-suppressor gene at 11q22–23 by LOH is not a very common event in the development of colorectal tumours, but may be biologically significant if accompanied by chromosome 14 deletions.

Colorectal carcinogenesis is a multistep process involving both the activation of proto-oncogenes and the inactivation of tumour-suppressor genes. The current model originally described by Fearon and Vogelstein (1990) identifies four main mutations, which include *ras* oncogene point mutations and allelic deletions and point mutations of the tumour-suppressor genes *APC*, p53 and *DCC* on chromosomes 5, 17 and 18 respectively. However, some adenomas have been identified which contain all four of these mutations but have not progressed to carcinoma, suggesting that additional genetic events are necessary for the transition from adenoma to carcinoma. In addition, frequent LOH has also been reported in colorectal tumours on chromosome 8 (Fujiwara *et al.*, 1993) and 14 (Young *et al.*, 1993).

Tumour-suppressor genes usually function in a recessive manner and therefore require both copies to be inactivated for tumour progression to occur (Knudson *et al.*, 1971). Loss of an allele is a common mechanism of inactivation, and hence detection of LOH is an important tool in identifying regions of the genome which may contain a tumour-suppressor gene. Chromosome 11 was considered a candidate for harbouring a tumour-suppressor gene because of cytogenetic analyses on colorectal cancers which have found frequent deletions in the long arm of chromosome 11 (Muleris *et al.*, 1990; Konstantinova *et al.*, 1991). In addition, a recent study of 39 human colorectal carcinomas by Keldysh *et al.* (1993) identified 11q deletions in 59% by restriction fragment length polymorphism (RFLP) and cytogenetic analyses. The smallest region of overlap (SRO) of these deletions was mapped to the region of 11q22–23. We have examined 101 colorectal cancers for allelic loss at the *DRD2* gene located at 11q22–23 in order to evaluate the findings of Keldysh *et al.* in a larger series.

### Materials and methods

### *Patient samples and cell lines*

Samples were obtained from 126 patients with sporadic colonic neoplasia during surgery or colonoscopy and purified macroscopically by a pathologist. Germline samples for each patient were obtained from normal colonic mucosa or peripheral blood leukocytes.

Correspondence: C.E. Gustafson.
Received 28 October 1993; and in revised form 9 February 1994.

The following cell lines were used in this study: HT-29, CaCo-2, Lisp-1, SW480, SW620, LIM1215, LIM1863, LIM2405, LIM2412, LIM1899, LIM2099, HCT-116, T84, LS174-T, KM12-SM, LS1034, LS411N, SW1116, LS513 and LoVo. Cell lines were cultured in RPMI-1640 medium or Dulbecco's modified Eagle medium (Gibco), supplemented with 10% fetal calf serum. DNA extraction from both patient material and cell lines was essentially as described by Miller *et al.* (1988).

### *PCR amplification reactions*

PCR amplification of the TG microsatellite in the *DRD2* locus was performed using the primers 509 and 419 (Hauge *et al.*, 1993) and produced a four-allele polymorphism with bands of 80, 82, 84 or 86 bp in size. In a total volume of 10 µl, 50 ng of genomic template DNA was amplified with 0.5 µM primers, 1.5 mM magnesium chloride (Promega), 1 × PCR buffer (Promega), 200 µM dTTP, dGTP, dCTP, 0.5 units of *Taq* polymerase (Promega), and 0.1 µl of [$^{35}$S]dATP (12.5 mCi, Amersham). Amplification reactions were carried out in an ITC100 thermal cycler (Bartelt Industries, Melbourne, Australia) as follows. An initial 4 min denaturation at 94°C was followed by 25 cycles of 40 s at 94°C, 30 s at 56°C and 20 s at 74°C, with a 3 min final extension at 74°C. PCR products were electrophoresed on a 5% polyacrylamide gel for 2 h. The gels were fixed, dried, then exposed to either Kodak X-OMAT film or a phosphor screen. This screen was scanned by the phosphorimager (Molecular Dynamics) after a 6–12 h exposure, and the autoradiographs were scanned by a densitometer (Molecular Dynamics) after a 24 h exposure. Resultant images were analysed using the computer software ImageQuant.

### *Analysis of LOH*

Analysis of informative cases for LOH was accomplished by quantitating the density of bands produced by [$^{35}$S]dATP incorporation during PCR amplification. LOH was calculated by a modification of the method by Solomon *et al.* (1987), in which a ratio of allelic imbalance of less than 0.75 or greater than 1.25 was considered to show LOH. Analysis of 2 × 2 tables was carried out using a two-tailed Fisher exact test, and trend analysis performed by the Wilcoxon's rank-sum test. The Mantel–Haenszel inference was used to stratify for stage.

### Results

Of the 126 patients analysed by PCR for microsatellite polymorphism at the *DRD2* locus at 11q22-23, 84 (66.7%) were informative. From these 84 informative patients, 101 samples (consisting of 30 adenomas, 68 carcinomas and three metastases) were analysed for LOH, which was demonstrated in a total of 30/101 (29.7%) informative lesions. Of 68 carcinomas tested, 23 (33.8%) showed allelic loss. This is significantly lower than the frequency of 11q cytogenetic deletions ($P = 0.0239$) and the combined frequency of cytogenetic deletions and RFLP allelic losses ($P = 0.0151$) reported by Keldysh *et al.* (1993). However, our low frequency of LOH does not differ significantly ($P = 0.0778$) from the figures that Keldysh *et al.* obtained by RFLP analysis alone.

Of 30 adenomas tested, five (16.7%) showed allelic loss. The mean size of adenomas not showing LOH was 1.1 cm, while that of adenomas showing LOH was slightly less than 1 cm. Of the nine patients from whom both an adenoma and a carcinoma were tested, one showed LOH in both the adenoma and carcinoma, two showed LOH in the carcinoma only, and the remaining six had no LOH in either the carcinoma or the adenoma. Metastatic tissue was examined from three patients and two showed allelic loss. For one of these two, primary carcinoma as well as metastatic tissue was available, and this also showed LOH. The one metastatic sample which showed no LOH had no loss in the primary tumour either. Of the 34 samples showing LOH, the larger allele was lost in 58% of cases, while the smaller was lost in 42% of cases. This is not significantly different from the expected ratio of 1:1 ($P = 0.62$) and indicates that the LOH was not an artifact caused by incomplete amplification of the larger alleles (Figure 1).

A summary of the histological and clinical features of the carcinomas tested is given in Table I. No significant associations were found between LOH on chromosome 11 and location ($P = 0.997$), Dukes' stage ($P = 0.881$), differentiation status of the tumour ($P = 0.922$) or sex of the patient ($P = 0.254$). The average age of patients in whose tumours allelic loss was demonstrated was 68.8 years compared with 68.3 years in those who did not exhibit LOH. Only one carcinoma with LOH was mucin producing. A further three mucin-producing tumours did not show LOH.

K-*ras* mutation and LOH data for nine other chromosome arms (1p, 1q, 5q, 8p, 14q, 17p, 17q, 18q, 22q) were available for these tumours (Young *et al.*, 1993; J. Young *et al.* submitted). There was no significant association between K-*ras* mutation or LOH at any of these sites and chromosome 11 (data not shown), with the exception of chromosome 14 (Young *et al.*, 1993). LOH on chromosomes 11 and 14 in carcinomas (Table II) was significantly associated ($P = 0.0007$), and this association was maintained when the tumours were stratified for Dukes' stage ($P = 0.028$).

Twenty cell lines established from human colorectal cancers were also analysed at the *DRD2* locus. While corresponding germ-line material was not available for comparison, 18/20 (90%) were heterozygous, therefore a maximum of 2/20 (10%) could have undergone allelic loss at this locus.

**Figure 1** LOH at the *DRD2* locus in colonic tumours showing loss of the 80 bp allele in the tumour of patient B.K. and of the 86 bp allele in the tumour of patient D.P.

### Discussion

A high rate of LOH in the region of chromosome 11 would suggest the existence of a putative tumour-suppressor gene which may be inactivated in human colorectal carcinoma. The rate of LOH on 11q in colorectal carcinomas found in our study (33.8%) was similar to the cytogenetic data found previously by Muleris *et al.* (1990), who found rearrangements in 28% of colorectal cancers examined. In contrast, Vogelstein *et al.* (1989) found LOH at the D11S144 locus at 11q22.3-23.3 in only 15% of tumours. Moreover, of 20 colorectal cell lines examined in this study only 10% were homozygous at this locus. Even if this was not due to natural homozygosity and had occurred through deletion of a second copy of a gene, 10% is a very low rate of LOH. These low rates of 10-33.8% LOH are somewhat inconsistent with those recently reported by Keldysh *et al.* (1993), who observed cytogenetic deletions or LOH at 11q in 23/39 (59%) of tumours. There are at least two possible explanations for the lower incidence of LOH in our study. Firstly, we examined a larger sample consisting of 101 informative cases instead of 39, and so the lower frequency that we report is probably more accurate. Secondly, our sample population was chosen completely randomly, with no enrichment of the sample population by prior knowledge of 11q chromosomal aberrations, whereas the sample that Keldysh *et al.* used included some tumours which were known by cytogenetic

**Table II** Chromosome 11 LOH associated with chromosome 14 LOH in carcinomas

| | Chromosome 14 Loss | Chromosome 14 No loss | Total |
|---|---|---|---|
| *Chromosome 11* | | | |
| Loss | 16 | 5 | 21 |
| No loss | 15 | 23 | 38 |
| Total | 31 | 28 | 59 |

**Table I** Histological and clinical characteristics of colorectal carcinomas examined for LOH on 11q

| | Location | | Dukes' stage | | | | Differentiation | | |
|---|---|---|---|---|---|---|---|---|---|
| | Right | Left | A | B | C | D | Poor | Moderate | Well |
| + LOH | 7 | 14 | 2 | 7 | 9 | 4 | 3 | 16 | 1 |
| − LOH | 15 | 29 | 4 | 24 | 10 | 7 | 7 | 35 | 2 |
| Total | 22 | 43 | 6 | 31 | 19 | 11 | 10 | 51 | 3 |
| LOH (%) | 32 | 33 | 33 | 23 | 46 | 36 | 30 | 51 | 33 |

analyses to have 11q abnormalities. These explanations are consistent with the fact that our results are not significantly different to the RFLP analysis that Keldysh *et al.* performed, whereas if the cytogenetic analyses were taken into account, significant differences were obtained. Keldysh *et al.* found non-significant associations between 11q LOH and (1) rectal location, (2) Dukes' A stage, (3) mucin production and (4) well-differentiated carcinomas. None of these associations was substantiated in this study. Keldysh *et al.* did not examine the *DRD2* locus itself, but it is located within the SRO (Eubanks *et al.*, 1992) that they defined by analysis of partial deletions. Keldysh *et al.* used Southern analysis of an RFLP to detect LOH, while we used PCR of a microsatellite. However, a direct comparison of these techniques in ten tumours of this sample for detecting LOH at p53 found them to give the same results (B. Leggett *et al.*, submitted). This is substantiated by the fact that the LOH rate at the p53 locus with a RFLP of low heterozygosity was 7/10 (70%) compared with 45/66 (68%) using a $(CA)_n$ repeat. There are now numerous reports of the use of microsatellite markers to detect LOH (Futreal *et al.*, 1992; Chenevix-Trench *et al.*, 1993; Linnenbach *et al.*, 1993). The major advantages are in saving of time and DNA, the high informativeness of the markers and the ability to use formalin-fixed material.

To confirm that contamination by normal mucosa did not result in an underestimation of the degree of allelic loss at this locus, the level of LOH on 11q was also shown to be low compared with the rate of 68% (45/66) observed in these carcinomas by PCR at the p53 locus, and 77% at the DCC locus by RFLP. However, 33.8% is probably above background (8–23% in this series) and, interestingly, does occur in 17% of adenomas in which the background rate is even lower. This level of LOH in adenomas suggests that inactivation of a gene at 11q22–23 may be an important early change in a subset of colon tumours. Similar frequencies of mutations in adenomas were observed at the 12th codon of K-*ras* in this sample (J. Young *et al.*, submitted), and in 17p and 18q class II adenomas by Vogelstein *et al.* (1988).

The possibility that a tumour-suppressor gene inactivated in colorectal cancer exists on 11q cannot be discounted simply because of the moderately low LOH rate observed in this study. Tanaka *et al.* (1991) found that human colon carcinoma cells into which a normal copy of chromosome 11 had been transferred exhibited a reduced tumour growth rate compared with that seen in the parental cells, although tumorigenicity was not reversed. This suggests that there may be a gene on chromosome 11 which has some effect on the rate of cell growth. Our data indicate that inactivation of a gene at 11q22–23 by LOH is not a very common event in the development of colorectal tumours but that it is significantly associated with LOH on chromosome 14. This indicates that chromosome 11 deletions may be important if they occur in combination with losses on chromosome 14.

We thank Drs Michael Ward, Russell Stitz and Alistair Cowen for providing samples, and Lesley Thomas and Ron Buttenshaw for technical assistance. Cell lines were obtained through the American Type Culture Collection, or kindly donated by Drs H. Lahm, J. Pugin and B. Whitehead. This work was supported by the Queensland Cancer Fund and the National Health and Medical Research Council of Australia.

### References

CHENEVIX-TRENCH, G., WICKING, C., BERKMAN, J., SHARPE, H., HOCKEY, A., HAAN, E., OLEY, C., RAVINE, D., TURNER, A., GOLDGAR, D., SEARLE, J. & WAINWRIGHT, B. (1993). Further localization of the gene for nevoid basal cell carcinoma syndrome (NBCCS) in 15 Australasian families: linkage and loss of heterozygosity. *Am. J. Hum. Genet.*, **53**, 760–767.

EUBANKS, J.H., DJABALI, M., SELLERI, L., GRANDY, D.K., CIVELLI, O., MCELLIGOTT, D.L. & EVANS, G.A. (1992). Structure and linkage of the D2 dopamine receptor and neural cell adhesion molecule genes on human chromosome 11q23. *Genomics*, **14**, 1010–1018.

FEARON, E.R. & VOGELSTEIN, B. (1990). A genetic model for colorectal tumorigenesis. *Cell*, **61**, 7559–7567.

FUTREAL, P.A., SODERKVIST, P., MARKS, J.R., IGLEHART, J.D., COCHRAN, C., BARRETT, J.C. & WISEMAN, R.W. (1992). Detection of frequent allelic loss on proximal chromosome 17q in sporadic breast cancer using microsatellite length polymorphisms. *Cancer Res.*, **52**, 2624–2627.

FUJIWARA, Y., EMI, M., OHATA, H., KATO, Y., NAKAJIMA, T., TAKESADA, M. & NAKAMURA, Y. (1993). Evidence for the presence of two tumor suppressor genes on chromosome 8p for colorectal carcinoma. *Cancer Res.*, **53**, 1172–1174.

HAUGE, X.Y., GRANDY, D.K., EUBANKS, J.H., EVANS, G.A., CIVELLI, O. & LITT, M. (1991). Detection and characterization of additional DNA polymorphisms in the dopamine D2 receptor gene. *Genomics*, **10**, 527–530.

KELDYSH, P.L., DRAGANI, T.A., FLEISCHMAN, E.W., KONSTANTINOVA, L.N., PEREVOSCHIKOV, A.G., PIEROTTI, M.A., PORTA, G.D. & KOPNIN, B.P. (1993). 11q deletions in human colorectal carcinomas: cytogenetic and restriction fragment length polymorphism analysis. *Genes Chrom. Cancer*, **6**, 45–50.

KNUDSON, A.G. (1971). Mutation and cancer: statistical study of retinoblastoma. *Proc. Natl Acad. Sci. USA*, **68**, 820–923.

KONSTANTINOVA, L.N., FLEISCHMAN, E.W., KNISCH, V.I., PEREVOSCHIKOV, A.G. & KOPNIN, B.P. (1991). Karyotype peculiarities of human colorectal adenocarcinomas. *Hum. Genet.*, **86**, 491–496.

LEGGETT, B.A., YOUNG, J.P., BUTTENSHAW, R., THOMAS, L.R., CHENEVIX-TRENCH, G., SEARLE, J. & WARD, M. (1994). Colorectal carcinomas show frequent allelic imbalance on the long arm of chromosome 17 with evidence for a specific target region. *Cancer Res.* (Submitted).

LINNENBACH, A.J., PRESSLER, L.B., SENG, B.A., KIMMEL, B.S., TOMASZEWSKI, J.E. & MALKOWICZ, S.B. (1993). Characterization of chromosome 9 deletions in transitional cell carcinoma by microsatellite assay. *Hum. Mol. Genet.*, **2**, 1407–1411.

MILLER, S.A., DYKES, D.D. & POLESKY, H.F. (1988). A simple salting out procedure for extracting DNA from human nucleated cells. *Nucleic Acids Res.*, **16**, 1215.

MULERIS, M., SALMON, R.-J. & DUTRILLAUX, B. (1990). Cytogenetics of colorectal adenocarcinomas. *Cytogenet. Cell Genet.*, **46**, 143–156.

SOLOMON, E., VOSS, R., HALL, V., BODMER, W.F., JASS, J.R., JEFFREYS, A.J., LUCIBELLO, F.C., PATEL, I. & RIDER, S.H. (1987). Chromosome 5 allele loss in human colorectal carcinomas. *Nature*, **328**, 616–619.

TANAKA, K., OSHIMURA, M., KIKUCHI, R., SEKI, M., HAYASHI, T. & MIYAKI, M. (1991). Suppression of tumorigenicity in human colon carcinoma cells by introduction of normal chromosome 5 or 18. *Nature*, **349**, 340–342.

VOGELSTEIN, B., FEARON, E.R., HAMILTON, S.R., KERN, S.E., PREISINGER, A.C., LEPPERT, M., NAKAMURA, Y., WHITE, R., SMITS, A.M. & BOS, J.L. (1988). Genetic alterations during colorectal-tumor development. *N. Engl. J. Med.*, **319**, 525–532.

VOGELSTEIN, B., FEARON, E.R., KERN, S.E., HAMILTON, S.R., PREISINGER, A.C., NAKAMURA, Y. & WHITE, R. (1989). Allelotype of colorectal carcinomas. *Science*, **244**, 207–211.

YOUNG, J., LEGGETT, B., WARD, M., THOMAS, L., BUTTENSHAW, R., SEARLE, J. & CHENEVIX-TRENCH, G. (1993). Frequent loss of heterozygosity on chromosome 14 occurs in advanced colorectal carcinomas. *Oncogene*, **8**, 671–675.

YOUNG, J., SEARLE, J., BUTTENSHAW, R., THOMAS, L., WARD, M., WILLIAMS, G., YOUNG, B., LEGGETT, B., CHENEVIX-TRENCH, G. An augmented model of colorectal tumour development. *J. Natl Cancer Inst.* (Submitted).